# Salmonella Septic Arthritis and Bacteremia in a Patient With Poorly Controlled Diabetes

**DOI:** 10.7759/cureus.20465

**Published:** 2021-12-16

**Authors:** Kai-Ming Chang, Gabriel Karkenny, Robin Koshy

**Affiliations:** 1 Division of Infectious Diseases, Department of Medicine, Donald and Barbara Zucker School of Medicine at Hofstra/Northwell Health, Manhasset, USA

**Keywords:** case report, bacteremia, septic arthritis, diabetes, salmonella

## Abstract

*Salmonella* belongs to the *Enterobacteriaceae* family and is a frequent gastroenteritis pathogen when the food is not well handled. We present a case of indolent septic arthritis of the knee secondary to *Salmonella* bacteremia and uncontrolled diabetes. The knee effusion analysis showed a total nucleated cell count of 9206 cells/uL and no organism was seen under Gram stain. Both blood culture and synovial fluid culture later grew *Salmonella enterica* serovar Enteritidis*.* Meticulous workups revealed his previously undiagnosed and uncontrolled diabetes as the sole risk factor for developing severe salmonellosis. Serious non-typhoidal *Salmonella* infections often occur in immunocompromising states such as extreme age, HIV, malignancy, corticosteroid use, and rheumatologic disorders. Extraintestinal salmonellosis warrants surveillance for the aforementioned conditions. This case was unique in that septic arthritis and bacteremia due to *Salmonella* in a healthy man led to a diagnosis of uncontrolled diabetes. Like other bacterial septic arthritis, antimicrobial agents and proper drainage are the keys to treatment success. At least two weeks of antimicrobial therapy is needed for the treatment of Salmonella soft-tissue infection; however, therapy for four-six weeks might be necessary given the known persistence of *Salmonella *species at compromised sites.

## Introduction

*Salmonella* are Gram-negative bacteria within the family *Enterobacteriaceae *[1]. They are important human pathogens and are zoonotic in nature. Non-typhoidal *Salmonella* such as S. *enterica* ser. Enteritidis and S. enterica ser. Typhimurium can cause a wide range of diseases, including focal infections, gastroenteritis, bacteremia, and endovascular infections [2]. Most cases of *Salmonella* infection are foodborne and consist of self-limited gastroenteritis [2]. Focal infections such as septic arthritis, on the other hand, are much less common. Herein, we present a case of an indolent *Salmonella* septic arthritis (SSA) occurring secondary to *Salmonella* bacteremia, with the only risk factor being uncontrolled diabetes mellitus.

## Case presentation

**Figure 1:**
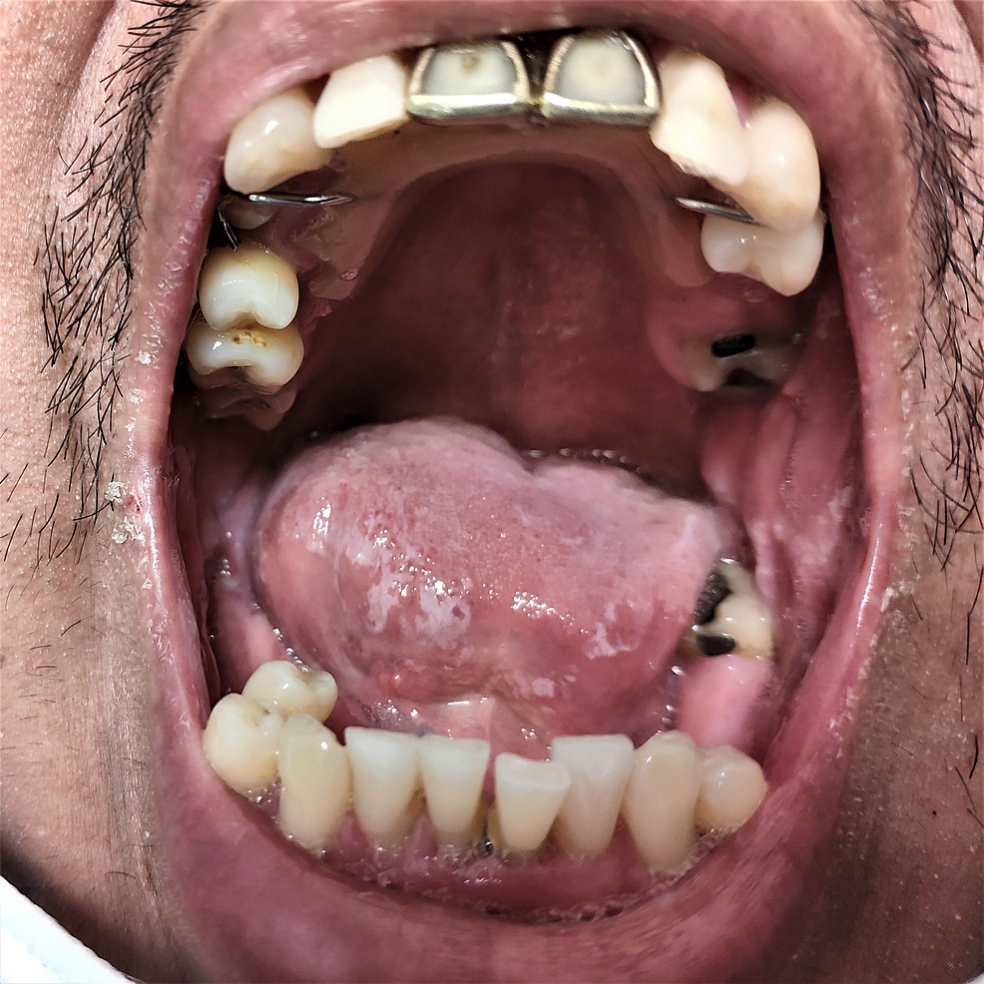
Oral thrush Poor dentition and oral thrush can be seen on the tongue, palate and buccal areas.

A 40-year-old Guatemalan man without any known past medical history presented to our emergency department (ED) in New York with right knee swelling, pain, and antalgic gait for two weeks. He denied subjective fever or chills. A set of peripheral blood cultures were obtained due to a temperature of 38.4 ^o^C in the ED. Serum white blood cell count was 12.81 K/uL (reference range, 3.8-10.5). He underwent right knee arthrocentesis, which revealed red color synovial fluid, with a total nucleated cell count of 9206 cells/uL (94% segmented granulocytes and 4% lymphocytes), a red blood cell count of 70000 cells/uL, and no crystal. Gram stain of the synovial fluid showed few polymorphonuclear (PMN) leukocytes per low power field but no organism was seen. Septic arthritis was not suspected by the ED so the patient was discharged home. Two days later, he was called back for admission because the anaerobic blood culture bottle grew Salmonella enterica serovar Enteritidis. It was susceptible to ampicillin, ceftriaxone, ciprofloxacin, and sulfamethoxazole-trimethoprim (SMZ-TMP). More detailed histories were obtained from the patient after admission. He worked as a landscaper. He was sexually active with one female partner. He denied any urinary discharge or dysuria. He recalled having a few days of diarrhea three weeks prior to admission. He denied eating any unusual food, with his typical meals involving home-cooked rice. Physical examination was notable for an overweight male with oral thrush (Figure 1).

**Figure 2:**
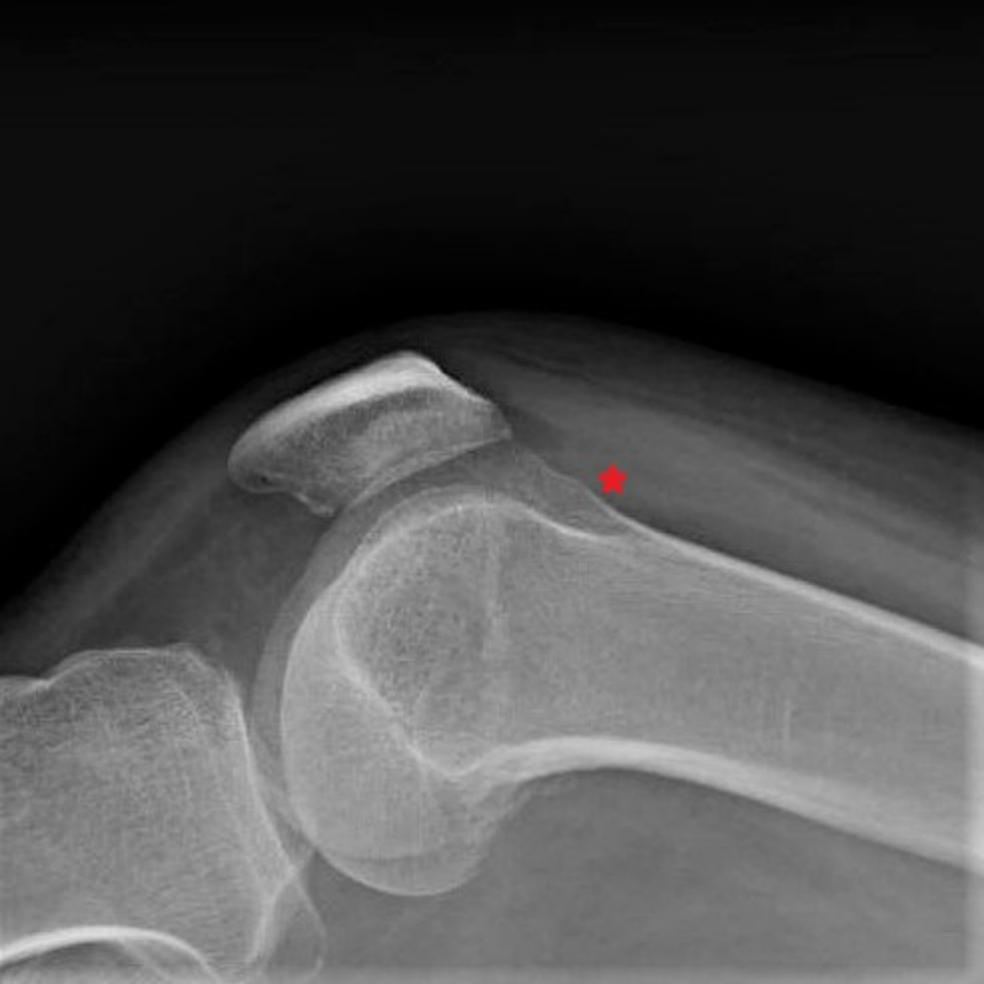
X-ray of the right knee (lateral view) The red asterisk shows the subtle area of effusion

The right knee was slightly warm and swollen compared to the left knee. However, there was no redness of the right knee. Heart, chest, and abdominal examinations were unremarkable. Human immunodeficiency virus (HIV) antigen/antibody/viral load and urine Chlamydia/Gonorrhea nucleic acid amplification test were negative. Serum hemoglobin A1c level was elevated at 8.4%. He had no anemia or personal history of sickle-cell disease. A plain radiograph of the right knee showed subtle effusion (Figure 2).

The computed tomography of the abdomen and pelvis did not show any acute findings. He was started on intravenous ceftriaxone 2 g every 24 hours and nystatin oral suspension four times per day. The synovial fluid culture grew *Salmonella* species after 96 hours of incubation. He underwent right knee aspiration again, and the culture did not grow any bacteria. Orthopedics evaluated and deemed the patient did not require further washout. Given the good tissue penetration property of SMZ-TMP, he was discharged home with oral SMZ-TMP double strength (800-160 mg) two tablets twice daily to complete a four-week course for the treatment of *Salmonella* septic arthritis and bacteremia.

## Discussion

**Figure 3:**
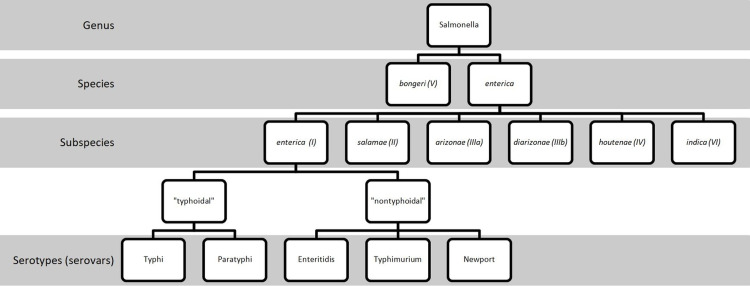
Nomenclature of Salmonella More than 2500 serovars were identified among seven subspecies of *Salmonella*. Only serovars Typhi, Paratyphi, Enteritidis, Typhimurium and Newport are listed here out of simplicity.

The genus *Salmonella*, a Gram-negative bacterium, is named in the honor of the American veterinarian Daniel Elmer Salmon [3]. It was first isolated in porcine intestines by Salmon’s assistant, Theobald Smith, in 1885 [3]. There are two species of *Salmonella*, *S. bongeri* (formerly subspecies V) and *S. enterica* (Figure 3) [1]. The latter can be further split into six subspecies that are designated by a Roman numeral and name (I, *enterica*; II, *salamae*; IIIa, *arizonae*; IIIb, *diarizonae*; IV, *houtenae*; and VI, *indica*) [1]. While subspecies II-IV are usual habitants of cold-blooded animals and the environment; subspecies I, *enterica* are the most accountable isolates in humans and domestic mammals [1]. The seven subspecies of *Salmonella* can be divided into more than 2500 serovars (serotypes) based on the combinations of flagellar antigens (H1 and H2) and lipopolysaccharide (O) or capsular polysaccharide (K) antigens [1]. Among subspecies, *S. enterica* subspecies *enterica* (subspecies I) accounts for approximately 60% of all serovars identified [1].

The most important serotypes of *S. enterica* that can cause human diseases are Typhi, Paratyphi, Enteritidis and Typhimurium [1,2]. Salmonella enterica serovar Typhi and Salmonella enterica serovar Paratyphi are typhoidal *Salmonella* that can cause enteric or typhoid fever. Non-typhoidal *Salmonella* such as S. *enterica* ser. Enteritidis and S. *enterica* ser. Typhimurium can cause a wide range of diseases, from gastroenteritis and focal infections (including meningitis, septic arthritis, osteomyelitis, cholangitis, and pneumonia), to bacteremia and endovascular infections such as arteritis and endocarditis [2,4]. It was noticed in 2016 that the Newport serotype had surpassed Typhimurium to become the second most frequently reported serotype in the United States [5]. Most cases of *Salmonella* infection are foodborne and may present with diarrhea. Risk factors for salmonellosis include extremes of age (including elderly and infants), alteration of the gut flora (as a result of antibiotics or surgery), diabetes [6], malignancy, rheumatologic disorders (e.g. systemic lupus erythematosus), biologics (e.g. infliximab or etanercept) [7], reticuloendothelial blockage (malaria, sickle-cell disease, or bartonellosis), human immunodeficiency virus (HIV) infection, corticosteroid use, or any other type of immunosuppression [2,4]. The use of proton pump inhibitors can decrease gastric acid secretion and increase susceptibility to *Salmonella* infection [8]. Diabetic patients may have impaired function of leukocytes, decreased gastric acid production, and decreased bowel motility that predisposes them to S. *enterica* ser. Enteritidis infection [6]. This is still unusual for a person like in our case who solely had uncontrolled diabetes but without other immunosuppressive conditions who presented with bacteremia that seeded to become a septic joint infection. Patients with extraintestinal *Salmonella* infections should be looked out for underlying conditions that might lead to an immunocompromised state as it may require a longer duration of treatment.

Non-typhoidal *Salmonella* can spread hematogenously to virtually any anatomical site and cause local infection [2]. *Salmonella* septic arthritis (SSA) is a rare complication of *Salmonella* infections, occurring in 0.27% [4,9]. Most cases of SSA are monoarticular and have no preexisting joint disease but can occur following trauma or in prosthetic joins [4,9-11]. The knee joint is the most commonly affected joint, followed by the hip and shoulder [4,9,10]. Bacterial arthritis usually causes an intense synovial fluid leukocytosis and is rarely less than 20000 cells/uL, while it often rises up to 50000 to 200000 cells/uL with greater than 90% of PMN [12]. In a review article by Cohen in 1987, the white blood cell count from joint fluid analysis ranged from 18500 to 169000 cells/uL [4]. Most patients had purulent joint fluid but about one-fifth of them had serous or serosanguinous fluid. Interestingly, the patient in our case seemed to have lower synovial PMN leukocytes than typically expected in bacterial septic joints. SSA, in contrast to *Staphylococcus aureus* joint infection, can have a subtle or lack of warmth around the joint, likely owing to the less virulent nature of *Salmonella* [13]. It may not cause restriction of joint motion or destruction of the joint space in the early infective period [13]. The relatively low white cell count in the joint in this case could make the infective process difficult to distinguish from reactive arthritis, which is an immune response to *Salmonella* that is associated with HLA-B27 antigen and is aseptic or sterile in nature [4]. Reactive arthritis is usually polyarticular and typically involves the knee, ankle, wrist, and small joints. Reiter syndrome should be suspected when a patient has reactive arthritis, urethritis, and conjunctivitis following dysentery [4]. The most important risk factor for *Salmonella* osteomyelitis is sickle-cell disease or hemoglobinopathy [4]. Osteomyelitis can occur concomitantly with septic arthritis; however, it was not seen in our case.

The treatment for *Salmonella* septic arthritis consists of aspiration of the joint, antibiotic therapy, and surgical drainage if the joint can’t be adequately aspirated [10]. The outcomes for SSA are typically better in comparison to septic arthritis caused by other Gram-negative bacteria [4]. Typically, antibiotic choices for non-typhoidal *Salmonella* include ampicillin, chloramphenicol, SMZ-TMP, third-generation cephalosporins, ciprofloxacin, and azithromycin. However, increasing antimicrobial resistance has been noted, and has been linked to agricultural use of antimicrobial agents [2]. Drug-resistant non-typhoidal *Salmonella* is a serious threat based on the 2019 Antibiotic Resistance Threats Report by the U.S. Centers for Disease Control and Prevention [14]. Ciprofloxacin non-susceptibility rates were on the rise and approaching 10% in 2017 [14]. Ceftriaxone resistance rate was 3% and the rate of decreased susceptibility to azithromycin was 1% [14]. Treatment duration for focal infections depends on whether source control can be achieved and the immune status of the host. In a normal host with surgically eradicated soft-tissue infection, a minimum of two weeks of antimicrobial therapy is suggested. However, four-six weeks of therapy is often advisable given the known persistence of *Salmonella* species at compromised sites [2]. Among a population of HIV-infected adults in Malawi who survived from non-typhoidal *Salmonella* bacteremia, 43% (19/44) had a first recurrence of bacteremia at 23-186 days [15]. Among these, 26% (5/19) developed multiple recurrences up to 245 days. Hence, prolonged treatment for immunocompromised hosts with non-typhoidal *Salmonella* bacteremia might be warranted despite no clear guideline to suggest treatment duration.

## Conclusions

This case report depicts a case of non-typhoidal *Salmonella* septic arthritis in a patient with underlying poorly controlled diabetes. *Salmonella* septic arthritis (SSA) should be differentiated from reactive arthritis. SSA can be treated with antibiotics and reactive arthritis is autoimmune in nature. The treatment for *Salmonella* septic arthritis is similar to Gram-negative bacterial septic arthritis, including antibiotics and drainage. Antimicrobial resistance in non-typhoidal *Salmonella* is on the rise and clinicians should be vigilant for joint infections by atypical pathogens such as *Salmonella* as the presentation might be indolent. Salmonellosis with no apparent cause should be evaluated for underlying immunocompromised conditions as the recurrence rate of *Salmonella* infection is high that might impact the treatment duration.

## References

[1] MacKenzie KD, Palmer MB, Köster WL, White AP (2017). Examining the link between biofilm formation and the ability of pathogenic Salmonella strains to colonize multiple host species. Front Vet Sci.

[2] Hohmann EL (2001). Nontyphoidal salmonellosis. Clin Infect Dis.

[3] Schultz M (2008). Theobald Smith. Emerg Infect Dis.

[4] Cohen JI, Bartlett JA, Corey GR (1987). Extra-intestinal manifestations of Salmonella infections. Medicine (Baltimore).

[5] (2021). National Enteric Disease Surveillance: Salmonella Annual Report, 2016. https://www.cdc.gov/nationalsurveillance/pdfs/2016-Salmonella-report-508.pdf.

[6] Telzak EE, Greenberg MS, Budnick LD, Singh T, Blum S (1991). Diabetes mellitus--a newly described risk factor for infection from Salmonella enteritidis. J Infect Dis.

[7] Sky K, Arroyo RA, Collamer AN (2013). Salmonella septic arthritis in a patient receiving etanercept: case report and review of the literature. Mil Med.

[8] Bavishi C, Dupont HL (2011). Systematic review: the use of proton pump inhibitors and increased susceptibility to enteric infection. Aliment Pharmacol Ther.

[9] David JR, Black RL (1960). Salmonella arthritis. Medicine (Baltimore).

[10] Morgan MG, Forbes KJ, Gillespie SG (1990). Salmonella septic arthritis: a case report and review. J Infect.

[11] Casado-Castillo F, Kobayashi T, Sekar P, Streit J, Molano De Pena I (2021). Prosthetic hip infection due to Salmonella enterica serovar Enteritidis. IDCases.

[12] Uygur E, Reddy K, Ozkan FÜ, Söylemez S, Aydin O, Senol S (2013). Salmonella enteridis septic arthritis: a report of two cases. Case Rep Infect Dis.

[13] Goldenberg DL, Reed JI (1985). Bacterial arthritis. N Engl J Med.

[14] (2021). Antibiotic Resistance Threats in the United States 2019. https://www.cdc.gov/drugresistance/pdf/threats-report/2019-ar-threats-report-508.pdf.

[15] Gordon MA, Banda HT, Gondwe M (2002). Non-typhoidal salmonella bacteraemia among HIV-infected Malawian adults: high mortality and frequent recrudescence. AIDS.

